# Gray matter asymmetry in asymptomatic carotid stenosis

**DOI:** 10.1002/hbm.25645

**Published:** 2021-09-09

**Authors:** Lei Gao, Yaqiong Xiao, Haibo Xu

**Affiliations:** ^1^ Department of Radiology Zhongnan Hospital of Wuhan University Wuhan City Hubei Province China; ^2^ Center for Language and Brain, Shenzhen Institute of Neuroscience Shenzhen China

**Keywords:** cortical organization, gradients, meta‐analysis, recall, vascular cognitive impairment, verbal memory

## Abstract

Even clinically “asymptomatic” carotid stenosis is associated with multidomain cognitive impairment, gray matter (GM) atrophy, and silent lesion. However, the links between them remain unclear. Using structural MRI data, we examined GM asymmetry index (AI) and white matter hyperintensity (WMH) in 24 patients with severe asymptomatic carotid stenosis (SACS), 24 comorbidity‐matched controls, and independent samples of 84 elderly controls and 22 young adults. As compared to controls, SACS patients showed worse verbal memories, higher WMH burden, and right‐lateralized GM in posterior middle temporal and mouth‐somatomotor regions. These clusters extended to pars triangularis, lateral temporal, and cerebellar regions, when compared with young adults. Further, a full‐path of WMH burden (X), GM volume (atrophy, M1), AI (asymmetry, M2), and neuropsychological variables (Y) through a serial mediation model was analyzed. This analysis identified that left‐dominated GM atrophy and right‐lateralized asymmetry in the posterior middle temporal cortex mediated the relationship between WMH burden and recall memory in SACS patients. These results suggest that the unbalanced hemispheric atrophy in the posterior middle temporal cortex is crucial to mediating relationship between WMH burden and verbal recall memories, which may underlie accelerated aging and cognitive deterioration in patients with SACS and other vascular cognitive impairment.

## Graphical abstract

Using a high‐dimensional voxel‐wise gray matter (GM) asymmetry algorithm, we identified rightward GM shifts in a language/speech pathway in severe asymptomatic carotid stenosis (SACS) patients. Further, the mediation analysis indicated that atrophy in the left posterior middle temporal gyrus (pMTG) and asymmetry in the right pMTG serially mediated the relationship between white matter hyperintensity (WMH) and verbal memory. Using metanalytical decoding and functional connectivity gradient map, we showed that the observed differences between SACS and controls in asymmetry index spanned across orofacial somatosensory/motor primary and temporal association cortices (two extreme ends of the principal gradient) and were in a potential “recall memory” network. These findings may facilitate a better understanding of the neuroanatomical basis of cognitive impairment in carotid stenosis.

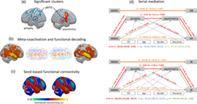

## 1 INTRODUCTION

Vascular cognitive impairment (VCI) is the second leading cause of dementia and increasingly affects global health and aging (Dichgans & Leys, 2017). One major contributor to VCI is advanced carotid stenosis. Increasing evidence suggests that even clinically “asymptomatic” carotid stenosis is associated with multidomain cognitive impairment (de Weerd et al., 2014). However, the neuroanatomical underpinning of this link remains unclear.

Carotid stenosis leads to hemodynamic consequences and silent lesions. Each has gained attention from the neuroimaging community and was often focused on perfusion or parenchymal changes (Avelar, D'Abreu, Coan, Lima, & Fernando, 2016; de Weerd et al., 2014; Kandiah, Goh, Mak, Marmin, & Ng, 2014). Of particular relevance to the neuroanatomical underpinning are findings that show symmetrical but stenosis‐ipsilateral dominated gray matter (GM) atrophy (Avelar et al., 2016; Gao, Ruan, Xiao, & Xu, 2021) and white matter hyperintensity (WMH) burden (Avelar et al., 2016; Gao et al., 2021), suggesting that unbalanced hemispheric damage (lateralization) contributes to the cognitive impairment in carotid stenosis.

So far, however, the links between hemispheric atrophy, WMH, and cognitive impairment remain unclear. It is empirically believed that precursor pathology to cerebrovascular disease is silent white matter damage, which promotes GM loss and consequent cognitive impairment. Clues could be found in neurodegenerative diseases, such as mild cognitive impairment (MCI) and Alzheimer's disease (AD). Recent evidence suggests that WMH burden predicts verbal memory deficits and is mediated by lateral temporal atrophy in AD (Swardfager et al., 2018). GM asymmetry, affected by hemispheric atrophy, tends to increase with age and alters in neurodegenerative diseases including MCI and AD (Minkova et al., 2017; Toga & Thompson, 2003). In addition, WMH burden is associated with brain atrophy and predicts dementia progression (Habes et al., 2016; Wen, Sachdev, Chen, & Anstey, 2006).

Advances in neuroimaging, meanwhile, have greatly enhanced our understanding regarding brain aging and macroscale organization. Multimodal imaging data sharing, for example, not only provides a basis for discovery, validation, and metanalytical synthesis, but also provides perspectives on cortical hierarchy centered on the default mode network (Margulies et al., 2016) and semantic representations of abstract cognitive operations (Huth, de Heer, Griffiths, Theunissen, & Gallant, 2016) in the human brain. These advances enable us to better understand multifactorial interplay underlying carotid stenosis from a neuroimaging framework.

**FIGURE 1:**
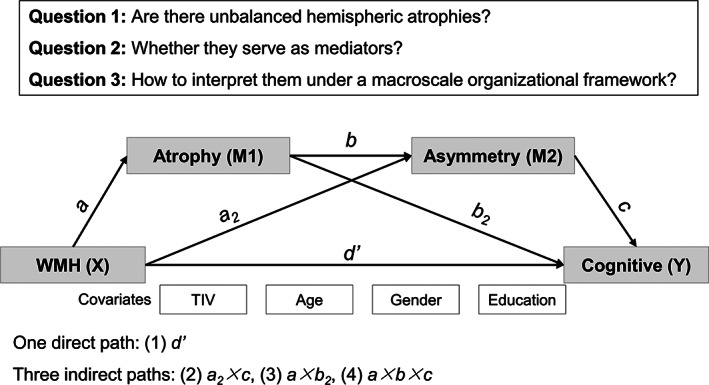
Schematic diagram of two‐mediator serial mediation analysis. TIV, total intracranial volume; WMH, white matter hyperintensity

In the present study, we sought to investigate the following key research questions (as illustrated in Figure 1) that related to cognitive decline in unilateral severe (>70%) asymptomatic carotid stenosis (SACS). We tested whether there are unbalanced hemispheric atrophies and whether these atrophies influence the relationship between WMH and the cognitive function. Further, we attempted to interpret these interrelationships under a macroscale cortical organization. These questions were investigated using two additional in‐house samples of young adults and elderly populations and a public life span dataset and metanalytical decoding. Specifically, we examined the voxel‐wise GM asymmetry and explored the relationships among hemispheric GM asymmetry, WMH, and neuropsychological performance in patients with SACS.

## 2 MATERIALS AND METHODS

This study was approved by the Medical Ethics Committee of Zhongnan Hospital of Wuhan University, and all participants gave written informed consent.

### 2.1 Participants

In this retrospective study, we collected data from 24 SACS patients and 24 healthy controls (HC) who were matched on comorbidities (underlying diseases, such as hypertension, diabetes, and hyperlipidemia) and demographics (i.e., age, gender, and education). These data have been partially reported previously (Gao et al., 2019). The SACS patients were: (a) aged 55–80 years; (b) right‐handed; (c) with unilateral internal carotid artery stenosis of 70–99% and contralateral stenosis ≤50%; (d) no posterior circulation diseases; (e) free of stroke, transient ischemic attack, or dementia; (f) with Modified Rankin Scale (Sulter, Steen, & De Keyser, 1999) ≤1; (g) with Mini‐Mental State Examination (MMSE; Tombaugh & McIntyre, 1992) score ≥ 26; (h) no severe systemic or neuropsychiatric diseases; (i) no contraindications for MRI; and (j) with education ≥6 years.

We included two in‐house validation datasets: 84 elderly HC aged 57.92 ± 4.94 years (39 females/45 males; matched with the HC sample on comorbidity and demographics) and 22 healthy young adults aged 26.95 ± 3.37 years (11 females/11 males). These independent datasets were collected using the same MRI scan protocols. Participants' demographic and clinical characteristics are summarized in Table S1.

Additionally, to trace the aging trajectories of the GM asymmetry, we analyzed publicly available data in the Southwest University Adult Lifespan Dataset (SALD; Wei et al., 2018; http://fcon_1000.projects.nitrc.org/indi/retro/sald.html). The SALD comprises a cohort of 494 cross‐sectional sample aged 19–80 years (307 females/187 males), including phenotypic information and multimodal MRI data.

### 2.2 Cognitive assessments

All participants (i.e., 24 SACS patients and 24 HC) completed a comprehensive cognitive battery within 7 days of the MRI scan, including (a) general cognitive assessment and screening using the MMSE (Tombaugh & McIntyre, 1992) and Montreal Cognitive Assessment Beijing Version (Nasreddine et al., 2005); (b) perceptual‐motor speed, memory, and visual scanning using the Digit Symbol (Strauss, Sherman, & Spreen, 2006); (c) verbal learning, memory, encoding, and retrieval using the Rey Auditory Verbal Learning Tests (RAVLT; Schmidt, 1996). A full description of the assessment procedures can be found in our recent publications (Gao et al., 2019; Gao et al., 2021; Wang et al., 2017; Xiao et al., 2018).

### 2.3 MRI protocols

MRI data were collected from all subjects (except the young adults) using a MAGNETOM Trio 3.0 T MR scanner (Siemens, Germany), including T1 anatomical (176 sagittal slices, 1‐mm in‐plane resolution) and T2‐weighted fluid attenuated inversion recovery (FLAIR; voxel size 0.5 × 0.5 × 1 mm, 160 slices) images covering the whole brain. Young adults were scanned using a dedicated 3.0 T Siemens PRISMA scanner (Siemens, Germany), with a 64‐channel head coil and similar parameters. Detailed descriptions of the MRI scan protocols have been reported in previous studies (Gao et al., 2019; Gao et al., 2021; Wang et al., 2017; Xiao et al., 2018).

### 2.4 Voxel‐based morphometry

Voxel‐based morphometry (VBM) was performed using a conventional method with Statistical Parametric Mapping (SPM12, http://www.fil.ion.ucl.ac.uk), Computational Anatomy Toolbox (CAT12.6, http://dbm.neuro.uni-jena.de/cat), and MATLAB 2013a (https://www.mathworks.com). The anatomical images were first visually inspected and then preprocessed with procedures including inhomogeneity correction, skull stripping, and registering to a diffeomorphic anatomical registration using lie algebra (DARTEL; Ashburner, 2007) template. The registered images were segmented into GM and white matter (WM) classes. The GM images were then spatially normalized into Montreal Neurological Institute (MNI) space (voxel‐size 1.5*1.5*1.5 mm^3^). The normalized images were modulated with a Jacobian determinant and smoothed with an 8 mm full‐width‐at‐half‐maximum (FWMH) Gaussian kernel.

### 2.5 GM asymmetry index (AI)

The AI was calculated following previous research (Kurth, Gaser, & Luders, 2015; Kurth, MacKenzie‐Graham, Toga, & Luders, 2015). The segmented GM and WM images from the VBM analysis were normalized and used to compute GM AI. Specifically, the normalized symmetrical GM and WM images were left–right flipped. The flipped and nonflipped GM and WM images were warped and normalized into the MNI space to create an analysis‐specific symmetric DARTEL template. A right‐hemispheric mask was then applied. The GM AI was calculated with the following formula:

$$\mathrm{AI}=2\frac{i3*\left(i1-i2\right)}{i1+i2}$$

where *i1* was the warped nonflipped GM images, *i2* was the warped flipped GM images, and *i3* was the right‐hemispheric mask. Negative AIs indicate leftward GM, while positive AIs indicate rightward GM. The AI images were smoothed using an 8‐mm FWMH smooth kernel.

Given the side of stenosis likely affects the direction of AI, we obtained individual signed *Z*‐score maps by subtracting the mean and divided by the standard deviation of the pooled HC (n = 24). The *Z‐*map of each patient was then thresholded (|*Z*| ≥ 3.1 and cluster size ≥10 voxels) within the right hemispheric mask, which resulted in an AI probability map (superimposed *Z* map).

### 2.6 White matter hyperintensity (WMH)

WMH size was calculated based on T1 and 3D T2‐FLAIR images using the Lesion Segmentation Tool (LST, www.applied-statistics.de/lst; Schmidt et al., 2012) as described in its tutorial and our recent publications (Gao et al., 2019; Gao et al., 2021). Lesions were segmented by the lesion growth algorithm with a default initial threshold (*κ* = 0.3; Schmidt et al., 2012).

### 2.7 Statistical analysis

Five SACS patients were excluded due to artifacts or low weight scores. Demographic and clinical data were contrasted between groups using two‐sample independent *t*‐tests for continuous variables and using *Chi*‐square tests for the categorical variable (i.e., gender) in SPSS 16.0 (SPSS Inc., Chicago, IL), with a significance level of *p* < .05. Imaging data were contrasted between groups using two‐sample *t*‐tests with total intracranial volume (TIV), gender, and age as covariates of no interest. The results were corrected for spatial extent across the GM (VBM) or right side hemisphere mask (AI) using a voxel‐wise threshold of *p* < .001 with a cluster‐wise family wise error (FWE) rate correction at *p* < .05.

We also examined Pearson's linear correlations between mean AI within each significant cluster, behavioral measurements, and WMH burden in SACS patients. All correlation results were corrected for multiple comparisons using the Bonferroni approach at a significance of *p* < .05.

### 2.8 Mediation analysis

A two‐step serial mediation analysis was conducted using Hayes's SPSS macro‐PROCESS (ver3.4, model 6; Hayes, 2018), with TIV, age, gender, and education as covariates of no interest. In the mediation model, the GM volume of the left posterior middle temporal gyrus (pMTG) served as the first mediator (M1), AI of the right pMTG as the second mediator (M2), and the predictor WMH burden (X) as affecting outcome recall memory (Y). There were four pathways: (1) one direct pathway *d'* and three indirect pathways including (2) *a*_*2*_ *× c*, (3) *a × b*_*2*_, and (4) *a × b × c* (Figure 1). We applied a bootstrapping method with 10,000 samples and a 95% confidence interval (CI) for indirect effect inference. A 95% CI that does not contain zero indicates a significant indirect effect (Hayes, 2018).

### 2.9 Metanalytical mapping and lifespan GM asymmetry trajectory

To verify the functional role and attached network system of the pMTG, we performed functional connectivity and metanalytical mapping with the bilateral pMTG seeds (MNI coordinates: ±58, −28, −2; spheres with 5 mm radius) from the *NeuroSynth* database (www.neurosynth.org; Yarkoni, Poldrack, Nichols, Van Essen, & Wager, 2011). Further, we performed a metanalysis with the term “recall” to obtain metanalytical activation maps.

Additionally, to delineate the developmental trajectory of AI over a broad age range of 18–80 years, we applied the same methodology in the SALD dataset and examined both linear and quadratic age effects.

## 3 RESULTS

As compared to HC, SACS patients showed significantly worse immediate (SACS: 31.0 ± 4.5; HC: 35.8 ± 5.6; SACS vs. HC: *p* < .005) and delayed recall memories (SACS: 4.6 ± 1.6; HC: 6.5 ± 1.1; SACS vs. HC: *p* < .001) but higher WMH loads (ratio between WMH size and TIV, WMH volumes, and WMH number; *p*s < .005). The two groups were comparable in gender, age, education, underlying diseases (hypertension, diabetes, and hyperlipidemia) and smoking (*p*s > .05). For details, please see Table S1.

### 3.1 Gray matter atrophies

**FIGURE 2:**
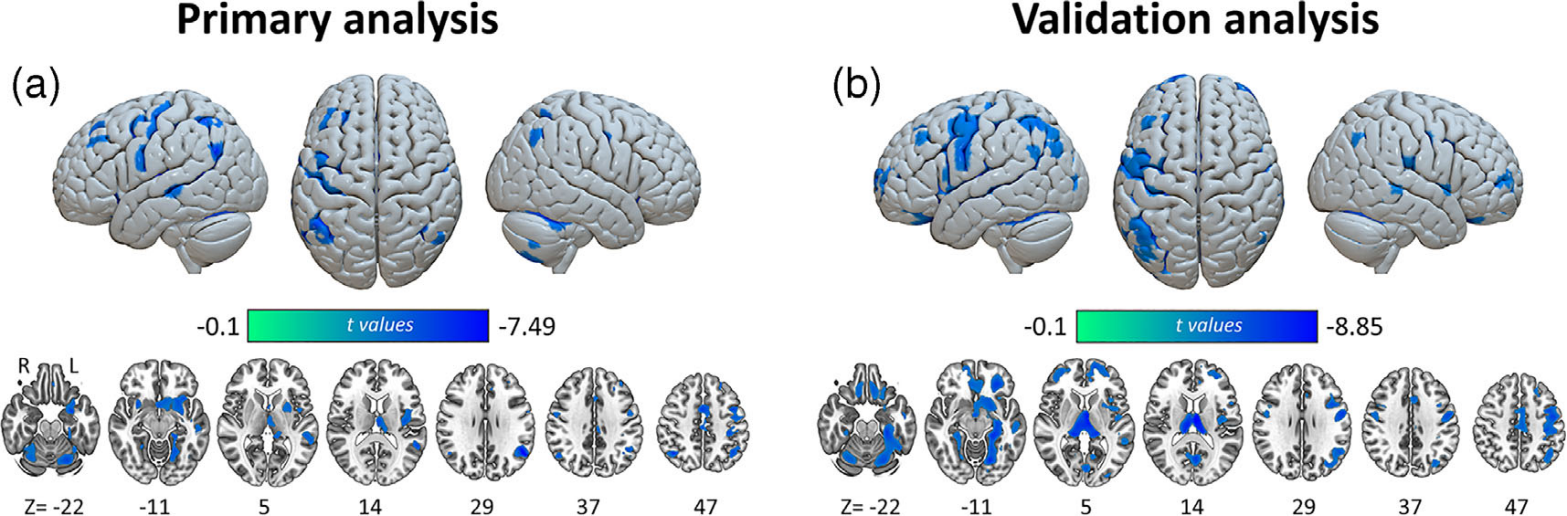
Group differences in VBM. (a) Primary and (b) validation results of group differences between SACS patients and controls in VBM, cluster‐level *p* < .05, FWE corrected. FWE, family wise error; L, left; R, right; SACS, severe asymptomatic carotid stenosis; VBM, voxel‐based morphometry. These maps were rendered by using the Surf Ice tool (https://www.nitrc.org/projects/surfice/) and MRIcroGL (https://www.nitrc.org/projects/mricrogl)

SACS patients showed significant GM atrophies in several clusters, including lateral temporal, frontal, basal ganglia/thalamus, insular/Rolandic operculum, amygdala, and parahippocampal regions in the left hemisphere; angular, somatomotor, and middle cingulate cortices spanning across both left and right hemispheres; and cerebelum_6, 8, and 7b in the right hemisphere (cluster‐level *p* < .05, FWE corrected; Figure 2a & Table S2). Similar results are shown in the validation analysis (Figure 2b & Table S3).

### 3.2 Gray matter asymmetries

**FIGURE 3:**
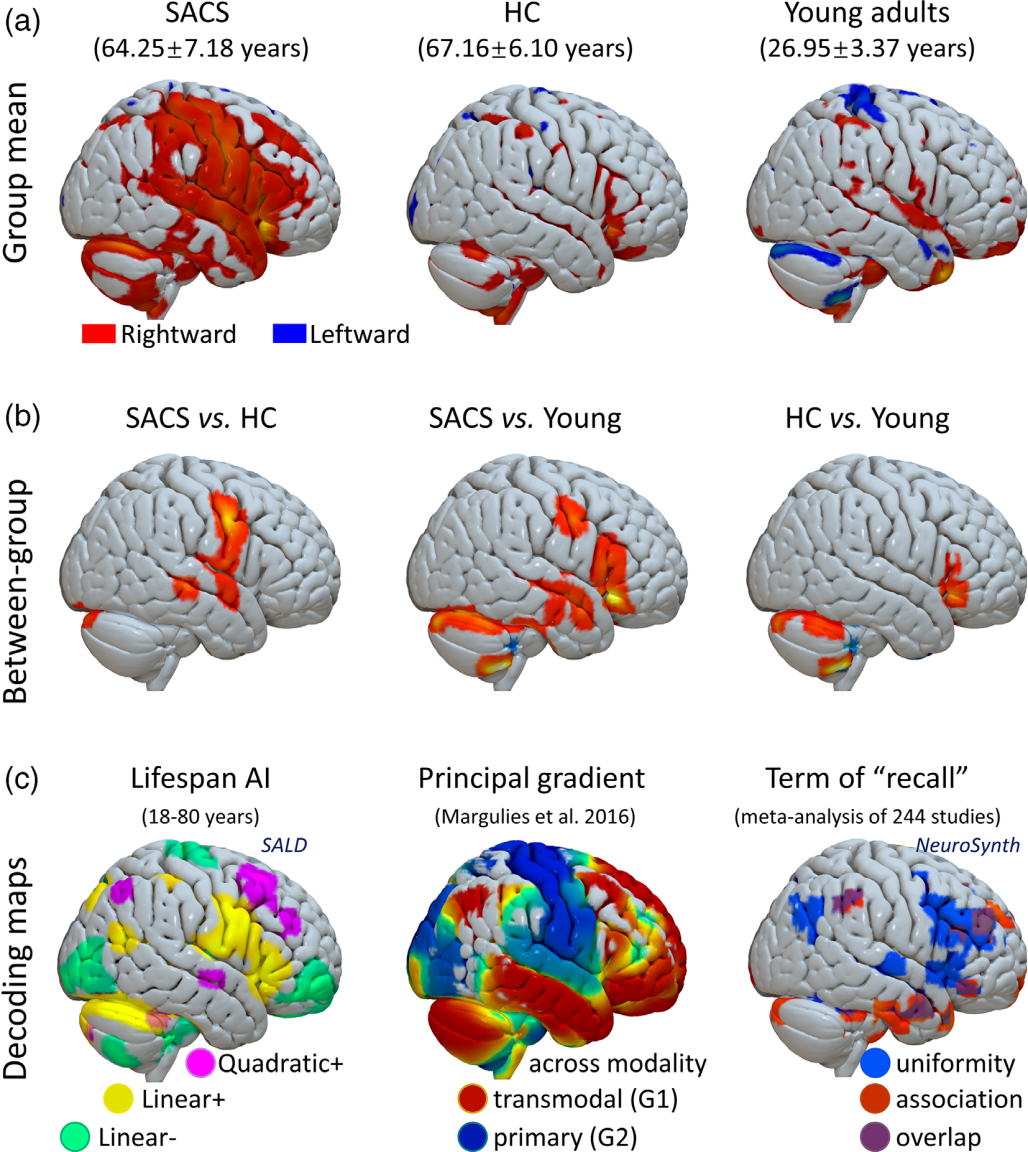
Gray matter asymmetries. (a) Mean AI distribution in SACS patients, elderly HC, and young adults. (b) Group comparisons in AI between SACS and elderly HC, between SACS and young adults, and between elderly HC and young adults. (c) Decoding maps. From left to right: (left) individual gray matter asymmetries of 483 subjects between the ages of 18 to 80 years with the SALD dataset, chronological age is both modeled as significantly linear age effect (positive linear with red‐yellow, and negative linear with green‐blue) and quadratic age effect (positive with violet; cluster‐level *p* < .05, FWE corrected); (middle) topography of principal gradient identified with functional connectivity from Margulies et al. (2016)) and *Neurovault* https://identifiers.org/neurovault.image:24346; (right) A metanalytic activation map related to “recall” (244 studies) from *NeuroSynth* (www.neurosynth.org; Yarkoni et al., 2011). G1, gradient 1; G2, gradient 2. FWE, family wise error; HC, healthy controls; SACS, severe asymptomatic carotid stenosis; SALD, Southwest University Adult Lifespan Dataset. These maps were rendered by using the Surf Ice tool (https://www.nitrc.org/projects/surfice/)

Group average of GM AI is shown in Figure 3a. In the primary analysis (19 SACS vs. 24 HC), we identified significantly rightward GM in clusters including hippocampus, superior/middle temporal gyri, pre/postcentral gyri (orofacial somatomotor), Rolandic operculum, fusiform, and thalamus in SACS (Table 1; Figure 3b, left panel). We also found similar results in the validation analysis (19 SACS vs. 84 independent HC; Table 1 & Figure S1).

**TABLE 1:** Regions showing significant group differences in gray matter asymmetry for both primary and validation analyses

Brain region	MNI coordinate			Extent	*T*	Top *NeuroSynth* terms
	*X*	*Y*	*Z*			
**Primary analysis**						
Precentral gyrus	60	0	38	453	5.76	Motor, force, movement, premotor, speech production, finger
Postcentral gyrus	64	2	14	453	4.26	Speech production, speech, SII, chosen, intervals, complexity
Thalamus	18	−28	4	719	5.06	Chosen, cerebellar, basal ganglia, ganglia
Middle temporal gyrus	58	−28	−2	296	4.98	Speech, auditory, listening, voice, language, comprehension
Cerebellum_Crus1	16	−88	−18	289	4.52	Visual stimuli, watching, autobiographical memory
Superior temporal gyrus	58	0	−10	85	4.47	Speech, auditory, listening, auditory cortex, voice, spoken
**Validation analysis**						
Thalamus	22	−24	6	2,459	8.44	Subcortical, basal ganglia, movements, motor, basal
Hippocampus	32	−12	−14	2,459	5.76	Episodic memory, expressions, positive negative, memories, faces
Caudate	8	20	2	2,459	4.62	Reward, striatal, monetary, punishment, incentive, anticipation
Postcentral	58	−2	34	1854	5.65	Sensorimotor, premotor, movements, aphasia, imitation, naming
Middle temporal gyrus	64	−6	12	1854	4.63	Speech, SI/II, nociceptive, listening, auditory, vocal
Inferior frontal gyrus	56	20	14	1854	4.51	Syntactic, language, linguistic
Cerebellum_Crus1	42	−72	−20	313	4.88	FFA, face, word form, visual, recognition, perception
Postcentral	38	−32	64	302	3.97	Motor, movement, hand, imagery, coordination, finger, execution

*Note*: The results were reported with a voxel‐wise *p* < .001 and a cluster‐level *p* < .05, FWE corrected. Peak foci were decoded with top functional terms (excluding anatomical terms) in *NeuroSynth* (www.neurosynth.org; Yarkoni et al., 2011).

Abbreviations: FFA, fusiform face area; MNI, Montreal Neurological Institute; SI, primary somatosensory cortex; SII, secondary somatosensory cortex; *T*, *t* statistical value; *x*, *y*, *z*, peak coordinate in the MNI standard space.

Additionally, we found significantly greater rightward AI in lateral temporal, orofacial somatomotor, pars triangularis (Broca's area), and cerebellar regions in SACS patients vs. young adults (Figure 3b, middle panel). The latter two clusters also consistently presented in the comparison between the HC and the young adults (Figure 3b, right panel).

In the SALD validation dataset, GM volumes in the inferior frontal/pars triangularis and orofacial somatomotor regions showed significantly age‐related rightward shifts (with both linear and quadratic age effects), but the pMTG did not show a trend of age‐related effect (Figure 3c, left panel).

### 3.3 Abnormal AI in SACS patients

**FIGURE 4:**
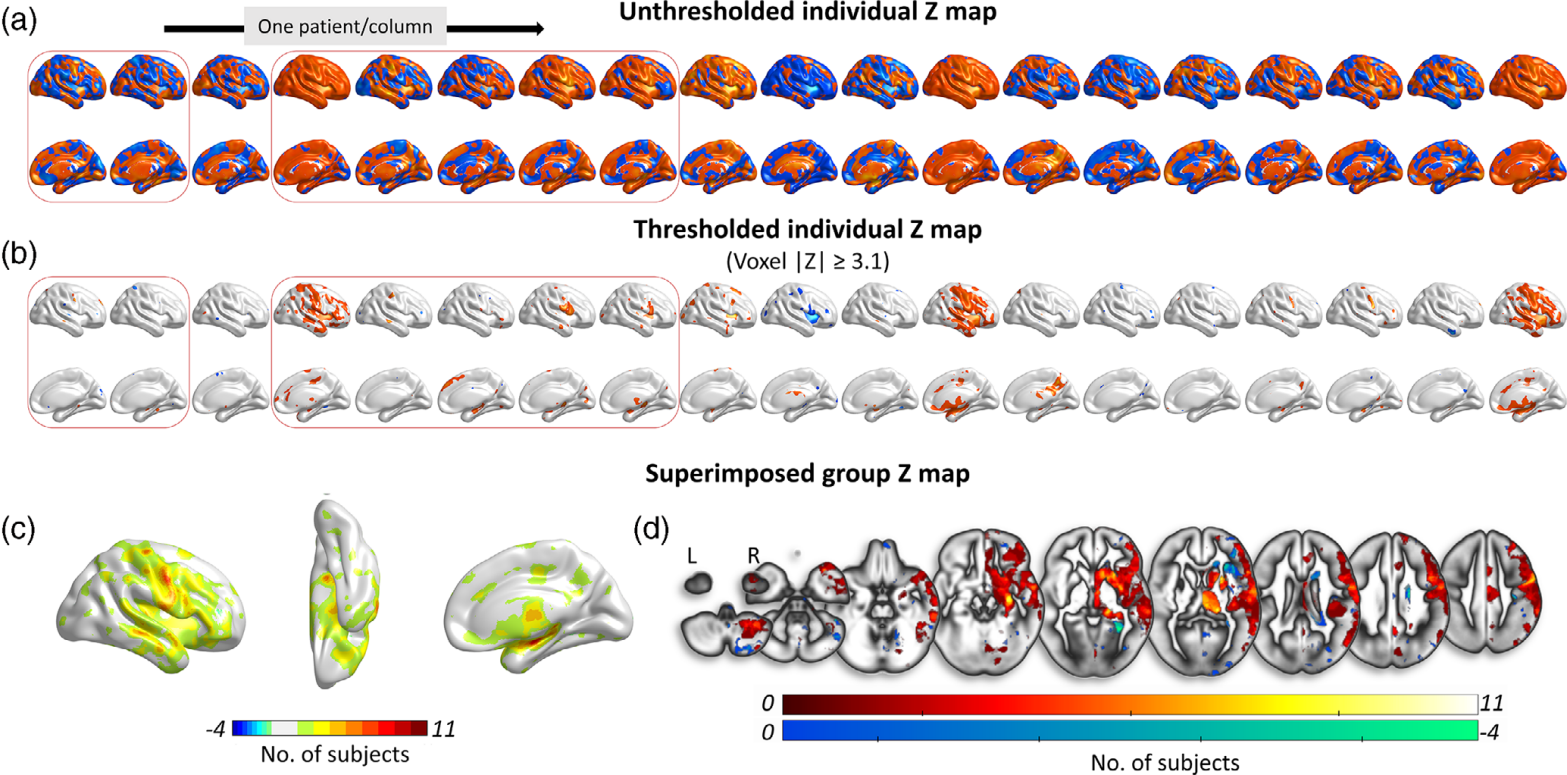
Individual AI maps and superimposed group AI maps in SACS patients. Individual differences in AI between SACS patients and pooled HC sample (*n* = 24). (a) Unthresholded *Z* maps of AI. (b) Thresholded *Z* maps of AI through comparing the ratio of [individual patient–group mean (HC)]/group std. (HC). The threshold level in B was set to *Z* value ≥3.1, corresponding to *p* < .001. The red boxes outline patients with left carotid stenosis. Superimposed probability maps based on the thresholded *Z* maps with both increased and decreased AI summed across the SACS patients after thresholded (|*Z*| ≥ 3.1) across SACS patients were displayed, in cortical (c) and slice (d) views. The color bar indicates number of patients with significant rightward (hot tone, up to 11) or leftward (cold tone, up to 4) AI in the same brain region. AI, asymmetry index; HC, healthy controls; SACS, severe asymptomatic carotid stenosis. These maps were visualized by using the BrainNet viewer (https://www.nitrc.org/projects/bnv/) and MRIcroGL (https://www.nitrc.org/projects/mricrogl)

As compared to HC (n = 24), SACS patients showed significantly different AI distributions (|*Z*| ≥ 3.1, cluster size >10 voxels; Figure 4a,b). Among 19 SACS patients, 11 of them showed significantly rightward AI in the lateral temporal, orofacial motor, thalamus, and parahippocampal regions, and 4 SACS patients showed significant leftward AI in insula (Figure 4c,d).

### 3.4 Associations between AI, behavioral, and lesion parameters in SACS patients

**FIGURE 5:**
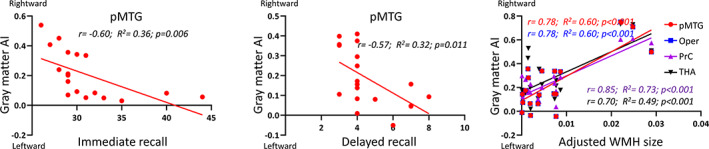
The associations between neuropsychological measures, WMH, and GM asymmetries in SACS patients. (a) Immediate recall was significantly negatively correlated with AI in the pMTG. (b) Delayed recall was significantly negatively correlated with AI in the pMTG. (c) Adjusted WMH size (i.e., the ratio of WMH size and total intracranial volume) was significantly positively correlated with AI in OPER, PrG, pMTG, and the thalamus. AI, asymmetry index; OPER, operculum insulae; pMTG, posterior middle temporal gyrus; PrG, precentral gyrus; SACS, severe asymptomatic carotid stenosis; THALA, thalamus; WMH, white matter hyperintensity

We found significantly negative associations between AI in the pMTG and both immediate recall (*r* [17] = −.60, *p* = .006) and delayed recall memories (*r* [17] = −.57, *p* = .011) in patients with SACS. Additionally, we found significantly positive associations between adjusted WMH size and AI in several regions, including the operculum insula (*r* [17] = .78, *p* < .001), the precentral gyrus (*r* [17] = .85, *p* < .001), the pMTG (*r* [17] = .78, *p* < .001), and the thalamus (*r* [17] = .70, *p* < .001; Figure 5).

### 3.5 Metanalytical decoding

**FIGURE 6:**
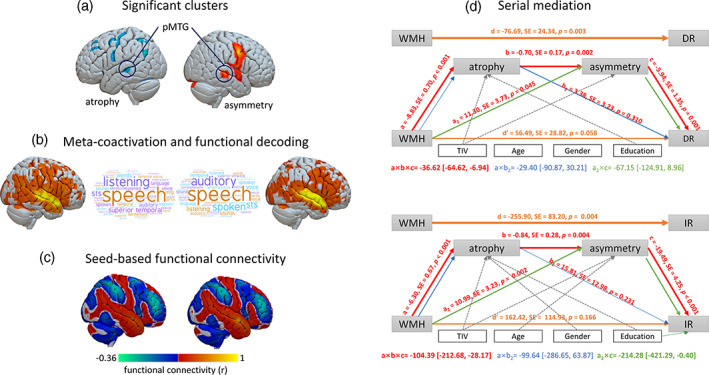
Functional decoding and serial mediation. (a) Significant clusters showing significant atrophy in the left pMTG and significantly rightward AI in the right pMTG. (b) Meta‐coactivation maps and word clouds showing top terms associated with the bilateral pMTG using the *NeuroSynth*. (c) Metanalytical functional connectivity maps with the bilateral pMTG as seed regions. (d) Path model tested in proof‐of‐principle sample (*n* = 43). Solid lines indicate significant pathways. Dash lines indicate nonsignificant pathways. For each connection, the coefficient (*a*, *b*, *c*, *a*_*2*_, and *b*_*2*_), its *SE*, and *p*‐value (*p*) are shown above the line. The mediation effect of the hypothesized serial pathway (*a × b × c*) and its 95% CI are shown in red, the mediation effect of left pMTG atrophy (*a*_*2*_ *× c*) in green, and the mediation effect of atrophy alone (*a × b*_*2*_) in blue. Direct effects are shown in orange; *d* is the direct effect adjusted only for covariates and *d'* is the direct effect adjusted for covariates and indirect effects. Covariate effects on mediators or outcomes with *p* < .05 are shown in gray. DR, delayed recall; IR, immediate recall; pMTG, posterior middle temporal gyrus; TIV, total intracranial volume. The brain maps were rendered by using the Surf Ice tool (https://www.nitrc.org/projects/surfice/)

We first identified the metanalytical co‐activation maps and functional roles of the pMTG. The pMTG showing significant atrophy and significant rightward AI (Figure 6a) was anchored to a mainly default mode network (and partially salience network) topography (Figure 6b,c), situated on a top end of the principal gradient (Figure 3c, middle panel). The pMTG was also involved in speech and semantic processing (Figure 6b).

We then decoded the functional co‐activation map with the term “recall”. The term “recall” (244 studies) from the *NeuroSynth* nicely outlined a topography similar to the resulting map from the group comparison between SACS and controls in AI (Figures 6b and 3c, right panel), and the principal gradient (Figure 3c, middle panel).

### 3.6 Serial mediation

#### 3.6.1 WMH to delayed recall

We found a significant total effect (path *d'*, *p* = .003), suggesting that increased WMH predicts decreased delayed recall memory (Figure 6d). In the path analysis, WMH negatively affected the left pMTG volume (*a* = −8.83, *p* < .001) but positively affected the right pMTG AI (*a*_*2*_ = 11.30, *p* = .045). The left pMTG volume significantly negatively impacted pMTG AI (*b* = −0.70, *p* = .002) and positively associated with delayed recall (*b*_*2*_ = 3.38, *p* = .310). The pMTG AI significantly negatively related to delayed recall (*c* = −5.94, *p* = .001). After controlling for the GM atrophy and AI, the direct effect of WMH on delayed recall was no longer significant (*d'* = 56.49, *p* = .058). Finally, the bootstrap procedure revealed a significant indirect effect that was significantly different from zero (*a × b × c* = −36.62, 95% CI [−64.62, −6.94; Figure 6d).

#### 3.6.2 WMH to immediate recall

Similar mediation effect was found in the immediate recall. The total effect of WMH on immediate recall was statistically significant (*d* = −255.90, *p* = .004; Figure 6d). After controlling for the GM atrophy and asymmetry, the direct effect of WMH on immediate recall was no longer significant (*d'* = 162.42, *p* = .166). Additionally, the indirect effects of WMH burden through GM asymmetry alone or together with atrophy were significantly different from zero (*a*_*2*_ *× c* = −214.28, 95% CI [−421.29, −0.40]; *a × b × c* = −104.39, 95% CI [−212.68, −28.17]; Figure 6d).

## 4 DISCUSSION

Using a high‐dimensional voxel‐wise GM asymmetry algorithm (Kurth, Gaser, & Luders, 2015; Kurth, MacKenzie‐Graham, et al., 2015), we identified rightward GM shifts in a language/speech pathway in SACS patients. Further, the mediation analysis indicated that atrophy in the left pMTG and asymmetry in the right pMTG serially mediated the relationship between WMH and verbal memory. Using metanalytical decoding and functional connectivity gradient map, we showed that the observed differences between SACS and controls in AI spanned across orofacial somatosensory/motor primary and temporal association cortices (two extreme ends of the principal gradient) and were in a potential “recall memory” network. These findings may facilitate a better understanding of the neuroanatomical basis of cognitive impairment in carotid stenosis.

### 4.1 Between‐group differences in AI

We replicated GM atrophies as shown in previous research in asymptomatic carotid stenosis (Avelar et al., 2016) and extended the analysis to the asymmetric atrophy (i.e., AI). This analysis identified pronounced rightward GM in SACS patients in a semantic/speech‐processing pathway that supports abstract cognitive functions including verbal learning and semantic memory, and primary functions including orofacial motor and speech (Skeide & Friederici, 2016).

These findings, corresponding with the carotid territory, suggest a left‐dominated atrophy and consequently right‐lateralized GM shifts. It is possibly manifested as hemispheric differences in vulnerability, hemodynamics, functional reserve, or silent lesions. Early dementia (Toga & Thompson, 2003) and neurodegenerative diseases (Minkova et al., 2017) are prone to left‐hemispheric structural and metabolic vulnerability. Silent microinfarct is a salient change in cerebral small vessel disease including asymptomatic carotid stenosis, and has been linked to (the progression of) cerebral atrophy (Blanco‐Rojas et al., 2013; Grau‐Olivares et al., 2007; Grau‐Olivares et al., 2010). An increasing body of evidence suggests that a main neural basis of cognitive dysfunction in carotid disease is the cerebral hemodynamic impairment. For example, the impaired cerebrovascular reactivity is associated with cognitive dysfunction (Lattanzi et al., 2018) and predicts cognitive functioning after carotid revascularization (Lattanzi et al., 2019), suggesting the contribution of cerebral hemodynamics as a potentially reversible determinant of cognitive dysfunction.

### 4.2 A path potentially mediating the relationship between WMH and recall memory

While asymptomatic carotid stenosis is known to be associated with atrophy and WMH, there is a gap among atrophy, WMH, and the cognitive function in individuals with SACS. Both clinical experience and recent evidence (de Weerd et al., 2014; Grau‐Olivares et al., 2010; Lal et al., 2017; Lattanzi et al., 2019; Lattanzi et al., 2020) suggest that these factors are likely to promote cognitive deterioration in SACS.

Our data demonstrated that atrophy and asymmetry in bilateral pMTG serially mediate the relationship between WMH and verbal memory, which provides insight into the link between neuroanatomical path and both carotid stenosis and verbal memory impairment. Similar to findings in AD patients (Swardfager et al., 2018), we found that WMH loads affect recall memory through AI alone or a combination with atrophy and asymmetry sequentially in SACS patients. These paths account for 19% (path *abc*) or 35% (path *a*_*2*_ *c*) variances of total effects in delayed recall, and 18% (path *abc*) or 37% (path *a*_*2*_ *c*) in immediate recall memory, respectively. The covariates of no interest, i.e., TIV, age, and education, differently interact with the mediators.

The roles of the pMTG in semantic retrieval, verbal memory, and multiple advanced network systems have been well documented in imaging (Davey et al., 2016; Karnath, 2001; Mesgarani, Cheung, Johnson, & Chang, 2014; Xu et al., 2015; Xu et al., 2019) and lesion (Leff et al., 2009; Martins et al., 2019) studies. Verbal learning and semantic retrieval are abstract cognitive functions in humans. They recruit perceptual input from auditory, somatomotor/sensory, and visual cortex. One example is the verbal learning task (Miotto et al., 2006; Miotto et al., 2014), in which subjects are presented with words auditorily and asked to recall the words as much as they can remember immediately or shortly. Completion of this task recruits dorsolateral prefrontal, orofacial sensorimotor, and inferior frontal regions, but suppresses activity in posterior temporal cortex (Miotto et al., 2006; Miotto et al., 2014). Many of these regions, however, were involved in the rightward AI in the SACS patients and in the “recall” topography, suggesting cortical reorganization at the macroscale.

### 4.3 Situating the AI differences on a macroscale hierarchical organization and aging trajectory

A recent milestone in the neuroimaging community is the discovery of hierarchical gradients embedded in the cerebral cortex (Margulies et al., 2016). These gradients define a geometric space of humans brain function and verify the Dual Origin Theory (Goulas, Margulies, Bezgin, & Hilgetag, 2019), in which the cerebral cortex is phylogenetically evolved from two distinct systems: amygdala‐centric and hippocampocentric systems. The hippocampocentric system is crucial for advanced cognitive functions and corresponds to the principal gradient. The lower end of the principal gradient anchors to the primary sensorimotor cortex and the top end to the default mode network (Raichle, 2015), and it engages in brain functions from simple perceptual and motor to progressively abstract function spectrums, including semantic retrieval and verbal memory.

Our data identified a link between the AI differences and the principal gradient, that is, the pMTG on the top end and auditory/orofacial motor regions on the lower end. Remarkably, bilateral pMTG serve as mediators in mediating the relationship between WMH and verbal memory and are also anchored to the default mode and somatomotor/sensory networks (the principal gradient) with metanalysis and seed‐based functional connectivity. Similarly, the metanalytical activation map with the term “recall” (244 studies) in *NeuroSynth* again coincides with the abovementioned topographies. These results suggest that SACS, a potential chronic hypoperfusion condition, may associate with reorganization in the principal gradient (i.e., the first two gradients).

An exception is that we did not find a significant mediating role of hippocampus in recall memory. It is possible due to the fact that hippocampus is relatively small and cannot survive after multiple corrections. On the other hand, from a network perspective (Fox, 2018), the pMTG is part of the extending network that involves in semantic processing and verbal memory.

Consistent with a recent report (Kong et al., 2018), we showed significant age‐related rightward shifts in inferior frontal and somatomotor regions in the SALD lifespan dataset. However, when using the bilateral pMTG as targets, we did not find significant linear nor quartic effects. These results suggest that SACS‐related AI asymmetric changes commix with both cognitive impairment and accelerated aging.

### 4.4 Clinical relevance

Considering the focal alterations in the semantic and speech circuit, our findings have some potential clinical applications. For example, intensive training (e.g., speech, aerobic exercises, and attentional training; Kurth, Gaser, & Luders, 2015; Kurth, MacKenzie‐Graham, et al., 2015) alters GM asymmetries and has shown therapeutic effects in primary progressive aphasia (Bonakdarpour et al., 2019). Recently, the orofacial area serves as a target of brain computer interface for neural decoding and speech synthesis (Anumanchipalli, Chartier, & Chang, 2019). These applications suggest that speech training may renormalize AI in patients with SACS.

### 4.5 Limitations

The present study has a few potential limitations that are worth noting. First, we analyzed changes in neuroanatomical pathways, a determinant in cognitive impairment of asymptomatic carotid stenosis and also changes in hemodynamics (Lattanzi et al., 2018;Lattanzi et al., 2019; Lattanzi et al., 2020); however, the effects on the neuroanatomical basis are still poorly understood, and future research is needed to further elaborate on this contribution. Second, the average age of the SACS subjects was >60 years, a stage where normal aging and pathologies (such as worsening hemodynamics and WMH burden) intricately intertwined, and thus longitudinal dataset might be needed to address how pathologies contribute to the morphological alterations. Future research should focus on neuroanatomical changes after carotid revascularization, and this is indeed one of the main goals of randomized clinical trials in this community (Howard et al., 2017; Marshall, Asllani, Pavol, Cheung, & Lazar, 2017). Increasing evidence suggests that revascularization can simultaneously improve cerebral hemodynamics, reverse cognitive deterioration (Lattanzi et al., 2019; Lattanzi et al., 2020), and even renormalize functional connectivity in the advanced networks (Porcu et al., 2019; Porcu et al., 2020). Third, we did not take into account the role of clinically silent lacunar infarcts in cognitive impairment in the sample of patients analyzed. This is important because more than half of the patients with a first‐ever lacunar stroke and without cognitive impairment show minor neuropsychological alterations (Blanco‐Rojas et al., 2013; Grau‐Olivares et al., 2007). These minor disturbances are mainly related to the presence of clinically silent lacunar infarcts at this early stage of the disease (Grau‐Olivares et al., 2010). Fourth, this study included independent samples across a wide age span. The structural data processing used a standard group‐wise symmetric template, which may bring age‐ (and disease‐) related minor mis‐registrations. Fifth, we included MRI data from two different scanners (i.e., Siemens Trio and Prisma). There might be differences caused by different scanners in image intensity/contrast that may lead to differences in tissue segmentations. Nevertheless, the calculation of AI is unlikely to be affected, as AI is a unitless metric. Finally, we did not include factors such as smoking, drinking, and comorbidities into the path‐analysis model, and thus it is unknown how these factors may affect the model. Future studies using more sophisticated models may establish these relationships.

## 5 CONCLUSIONS

In conclusion, SACS is associated with left‐dominated atrophy and consequently right‐lateralized GM shifts in the lateral temporal regions. Furthermore, unbalanced hemispheric atrophy in the lateral temporal region is crucial in mediating relationship between WMH burden and recall memory, which may underlie accelerated aging and cognitive deterioration in SACS and other VCI.

## AUTHOR CONTRIBUTIONS

**Lei Gao**: Collected data, designed the methods, performed analysis, and wrote the manuscript. **Yaqiong Xiao:** Revised the manuscript, performed the analysis, interpreted, and discussed the results. **Haibo Xu**: Conceived the study, interpreted and discussed the results, and revised the manuscript. All the authors contributed to the revision of the manuscript.

## Supporting information


**Appendix** **S1.** Supporting Information.

## Data Availability

The data that support the findings of this study are available on request from the corresponding author.
