# Supplementary material for: Gray matter asymmetry in asymptomatic carotid stenosis
Source: Hum Brain Mapp. 2021 Sep 9;42(17):5665–76. doi: 10.1002/hbm.25645 (PMC8559457; doi:10.1002/hbm.25645)
Supplement: Supplementary file 1 — Appendix S1. Supporting Information. [file HBM-42-5665-s001.docx]

**Table S1.** **Demographics and Clinical Characteristics.**

|  | **SACS (n=19)** | **HC (n=24)** | ***P value*** |
| --- | --- | --- | --- |
| Age (years) | 64.3 (7.2) | 67.2 (6.1) | 0.17 |
| Sex |  |  | 0.99^a^ |
| Male | 15 | 19 |  |
| Female | 4 | 5 |  |
| Education (years) | 9.6 (2.8) | 11.1 (3.5) | 0.12 |
| Hypertension | 17 (89%) | 18 (75%) | 0.23 |
| Diabetes | 4 (21%) | 4 (17%) | 0.71 |
| Hyperlipidemia | 9 (47%) | 11 (46%) | 0.92 |
| Smoke | 9 (47%) | 6 (25%) | 0.13 |
| Affected side | 7L/12R | - | ^-^ |
| MMSE | 26.8 (0.7) | 27.4 (0.7) | 0.015* |
| MoCA | 23.3 (1.2) | 24.2 (1.6) | 0.017* |
| Word fluency | 33.4 (6.2) | 37.1 (4.0) | 0.258 |
| Digit symbol substitution | 28.0 (4.7) | 31.5 (5.5) | 0.029* |
| Backwards digit-span | 5.8 (1.0) | 6.5 (0.9) | 0.042* |
| Forwards digit-span | 3.8 (0.8) | 4.5 (0.8) | 0.021* |
| Immediate recall | 31.0 (4.5) | 35.8 (5.6) | 0.004** |
| Delayed recall | 4.6 (1.6) | 6.5 (1.1) | <0.001*** |
| WMH corrected | 0.6 (0.7) | -0.6 (1.0) | <0.001*** |
| WMH size (ml) | 10.8 (3.5) | 1.6 (3.1) | <0.005** |
| WMH number | 13.4 (5.8) | 5.9 (6.3) | <0.001*** |

*Values are presented as mean (SD) or number (%). L, left; R, right; MMSE, Mini-Mental State Examination; MoCA, Montreal Cognitive Assessment; a, two-tailed Chi-squared test; WMH, white matter hyperintensity; WMH corrected, white matter hyperintensity (corrected by total cranial volume and log10 transformed); WMH size, white matter hyperintensity volumes; WMH number, number of WMH; SACS, severe asymptomatic carotid stenosis; HC, healthy controls.*

| **Brain region** | **MNI coordinate (mm)** | | | **Cluster size** | **T values** |
| --- | --- | --- | --- | --- | --- |
|  | **X** | **Y** | **Z** | **(voxels)** |  |
| Insula_L | -29.5 | 8.5 | -16.5 | 13345 | -4.282 |
| Caudate_L | -4.5 | 6.5 | -3.5 | 13345 | -4.472 |
| Temporal_Mid_L | -59.5 | -31.5 | -1.5 | 12806 | -7.4867 |
| Angular_L | -53.5 | -59.5 | 28.5 | 12806 | -7.4455 |
| Parietal_Inf_L | -48.5 | -52.5 | 55.5 | 12806 | -5.6523 |
| Angular_R | 41.5 | -59.5 | 46.5 | 1962 | -6.2561 |
| Supp_Motor_Area_R | 1.5 | 1.5 | 47.5 | 4013 | -5.9232 |
| Cingulate_Mid_R | 2.5 | -23.5 | 48.5 | 4013 | -5.3305 |
| Cingulate_Mid_L | -1.5 | 18.5 | 36.5 | 4013 | -4.6645 |
| Cerebelum_6_R | 32.5 | -56.5 | -27.5 | 20510 | -5.9223 |
| Cerebelum_8_R | 17.5 | -72.5 | -53.5 | 20510 | -5.8764 |
| Cerebelum_7b_R | 36.5 | -61.5 | -46.5 | 20510 | -5.8526 |
| Amygdala_L | -30.5 | -0.5 | -22.5 | 13345 | -5.7262 |
| Insula_L | -34.5 | 16.5 | -11.5 | 13345 | -5.3004 |
| Caudate_L | -7.5 | 16.5 | -7.5 | 13345 | -5.2882 |
| Precentral_L | -42.5 | 2.5 | 46.5 | 7732 | -5.6733 |
| Postcentral_L | -35.5 | -29.5 | 48.5 | 7732 | -5.3133 |
| Cerebelum_6_L | -21.5 | -66.5 | -24.5 | 14298 | -5.6236 |
| ParaHippocampal_L | -23.5 | -32.5 | -15.5 | 14298 | -5.5363 |
| Thalamus_L | -2.5 | -15.5 | 13.5 | 14298 | -5.2863 |
| Rolandic_Oper_L | -38.5 | -16.5 | 16.5 | 2389 | -5.1517 |
| Insula_L | -44.5 | 1.5 | 4.5 | 2389 | -4.6194 |
| Frontal_Sup_2_L | -24.5 | 36.5 | 46.5 | 903 | -5.0339 |
| Frontal_Mid_2_L | -44.5 | 29.5 | 40.5 | 903 | -4.466 |
| Precentral_R | 53.5 | -5.5 | 39.5 | 539 | -4.7982 |
| Rectus_L | -3.5 | 39.5 | -19.5 | 575 | -4.6099 |

**Table S2.** Between-group VBM results on primary analysis (SACS vs. 24 HC). The results were reported with voxel-wise *p* < 0.001 and FWE corrected at cluster-level *p* < 0.05. Abbreviations. MNI, Montreal Neurological Institute; x, y, z, peak coordinate in the MNI standard space; L, left; R, right.

| **Brain region** | **MNI coordinate (mm)** | | | **Cluster size** | **T values** |
| --- | --- | --- | --- | --- | --- |
|  | **X** | **Y** | **Z** | **(voxels)** |  |
| Thalamus_L | -5 | -11 | 11 | 28427 | 8.809 |
| Cerebelum_6_L | -23 | -69 | -15 | 28427 | 7.965 |
| Hippocampus_L | -18 | -33 | -2 | 28427 | 7.906 |
| Precentral_L | -57 | -3 | 30 | 4871 | 6.947 |
| Postcentral_L | -38 | -29 | 51 | 4871 | 6.934 |
| Frontal_Mid_2_L | -39 | 2 | 50 | 4871 | 6.553 |
| Angular_L | -53 | -57 | 27 | 2328 | 6.466 |
| Parietal_Inf_L | -44 | -60 | 50 | 2328 | 5.894 |
| Occipital_Mid_L | -35 | -74 | 27 | 2328 | 5.864 |
| Precentral_L | -44 | 11 | 32 | 731 | 6.314 |
| Front_Inf_Oper_L | -54 | 12 | 14 | 731 | 5.108 |
| Frontal_Mid_2_L | -41 | 33 | 23 | 731 | 4.946 |
| Temporal_Mid_L | -54 | -21 | -15 | 1598 | 6.208 |
| Cingulate_Mid_L | -5 | -23 | 45 | 1491 | 5.731 |
| Precentral_R | 45 | 8 | 32 | 155 | 5.690 |
| Precentral_R | 50 | -3 | 33 | 391 | 5.500 |
| Occipital_Mid_L | -33 | -90 | 3 | 92 | 5.343 |
| Frontal_Sup_2_L | -26 | 32 | 45 | 168 | 5.220 |
| SupraMarginal_R | 63 | -23 | 21 | 64 | 5.132 |
| Temporal_Inf_L | -42 | -63 | -6 | 99 | 5.081 |
| Lingual_L | -17 | -84 | -9 | 52 | 5.037 |
| Occipital_Mid_L | -27 | -87 | 26 | 71 | 5.037 |
| Postcentral_L | -59 | -21 | 17 | 62 | 4.929 |
| Temporal_Mid_R | 66 | -33 | -2 | 59 | 4.871 |

**Table S3.** Between-group VBM results on validation analysis (SACS vs. 84 independent HC). The results were reported with voxel-wise *p* < 0.001 and FWE corrected at cluster-level *p* < 0.05. Abbreviations. MNI, Montreal Neurological Institute; x, y, z, peak coordinate in the MNI standard space; L, left; R, right.


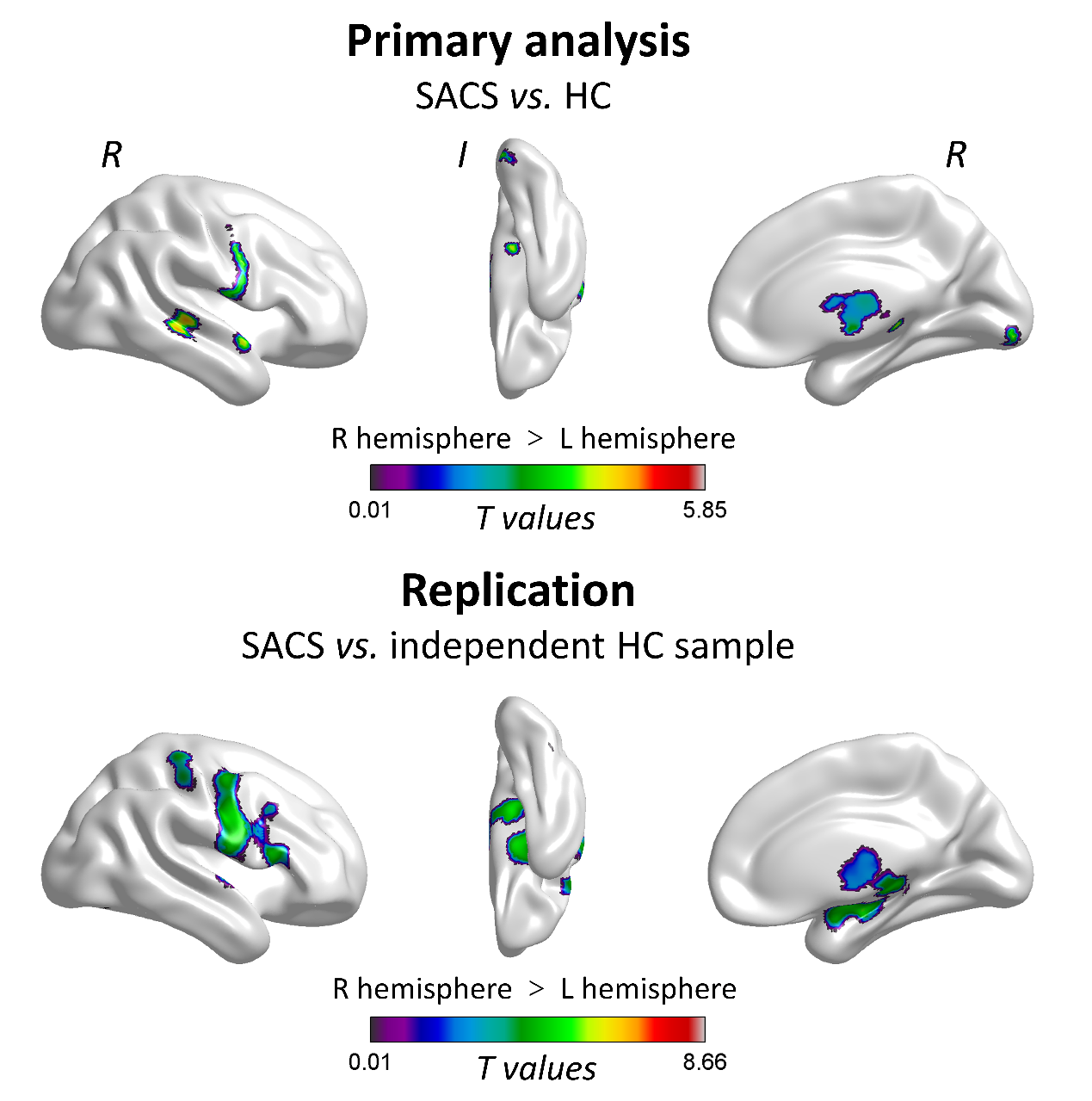


**Fig. S1. Between-group comparison on voxel-wise gray matter asymmetries.** Upper panel shows primary comparison of 19 SACS patients versus 24 HC. Lower panel shows validation comparison of 19 SACS patients and an independent sample (n=84) of HCs. Positive T value encoded in the colorbar denotes the extent of rightward AI in the patients with SACS. Since we did not find significant leftward AI in the patients, the negative T value in the colorbar was not shown. Abbreviations. L, left; R, right.
